# Potential of Adipose-Derived Mesenchymal Stem Cells and Skeletal Muscle-Derived Satellite Cells for Somatic Cell Nuclear Transfer Mediated Transgenesis in Arbas Cashmere Goats

**DOI:** 10.1371/journal.pone.0093583

**Published:** 2014-04-03

**Authors:** Yu Ren, Haiqing Wu, Yuzhen Ma, Jianlong Yuan, Hao Liang, Dongjun Liu

**Affiliations:** 1 Key Laboratory of Mammalian Reproductive Biology and Biotechnology Ministry of Education, Inner Mongolia University, Hohhot, Inner Mongolia, China; 2 Inner Mongolia People’s Hospital, Hohhot, Inner Mongolia, China; University of California, San Diego, United States of America

## Abstract

Somatic cell nuclear transfer is used to generate genetic models for research and new, genetically modified livestock varieties. Goat fetal fibroblast cells (gFFCs) are the predominant nuclear donors in Cashmere goat transgenic cloning, but have disadvantages. We evaluated the potential of goat adipose-derived mesenchymal stem cells (gADSCs) and goat skeletal muscle-derived satellite cells (gMDSCs) for somatic cell nuclear transfer, evaluating their proliferation, pluripotency, transfection efficiency and capacity to support full term development of embryos after additive gene transfer or homologous recombination. gADSCs and gMDSCs were isolated by enzyme digestion and differentiated into neurocytes, myotube cells and insulin-producing cells. Neuron-specific enolase, fast muscle myosin and insulin expression were determined by immunohistochemistry. Following somatic cell nuclear transfer with donor cells derived from gADSCs, gMDSCs and gFFCs, transfection and cloning efficiencies were compared. Red fluorescent protein levels were determined by quantitative PCR and western blotting. 5-Methylcytosine, H4K5, H4K12 and H3K18 were determined immunohistochemically. gADSCs and gMDSCs were maintained in culture for up to 65 passages, whereas gFFCs could be passaged barely more than 15 times. gADSCs and gMDSCs had higher fluorescent colony forming efficiency and greater convergence (20%) and cleavage (10%) rates than gFFCs, and exhibited differing H4K5 histone modification patterns after somatic cell nuclear transfer and in vitro cultivation. After transfection with a pDsRed2-1 expression plasmid, the integrated exogenous genes did not influence the pluripotency of gADSCs–pDsRed2-1 or gMDSCs–pDsRed2-1. DsRed2 mRNA expression by cloned embryos derived from gADSCs–pDsRed2-1 or gMDSCs–pDsRed2-1 was more than twice that of gFFCs–pDsRed2-1 embryos (*P*<0.01). Pregnancy rates of gADSCs–pDsRed2-1 and gMDSCs–pDsRed2-1 recipients were higher than those of gFFCs–pDsRed2-1 recipients (*P*<0.01). With their high proliferative capacity and transfection efficiency, gADSCs and gMDSCs are a valuable cell source for breeding new, genetically modified varieties of livestock by somatic cell nuclear transfer.

## Introduction

In recent years genetically engineered pigs have been produced for xenotransplantation [1] and cattle have been successfully produced by somatic cell nuclear transfer (SCNT) [2]. The breeding and application of new genetically modified varieties of Arbas cashmere goats with high economic value is strategically significant for modern animal husbandry.

The establishment of mouse embryonic stem cells (ESCs) has promoted the development of ESC-based gene targeting technology. Accurate and efficient gene knockout and knock in are now possible in mouse models. Because the isolation and culture of ESCs from large animals is relatively more difficult, there has been no successful long term culture of goat ESCs; thus, goat ESCs cannot be used in transgenic animal cloning research. In addition, SCNT using pools of stable transfected cell clones is an efficient means of producing transgenic goats with appropriate expression patterns [3], [4] and has facilitated the generation of goat models with inducible transgene expression [5]. Among the various parameters influencing the outcome of SCNT, including oocyte quality, embryo culture and recipient animal preparation, the type and quality of the nuclear donor cells are of vital importance [6].

Many primary cell lines established after passaging a primary culture have been used for SCNT in pigs, differing with respect to cell type, source organ, and the age and gender of the donor animal. Among these lines, mesenchymal stem cells have been precisely characterized [7], [8]. These cells have been tested for their efficiency in SCNT experiments in pigs, but further characterization of, for example, the specific cell types and their morphology, proliferation and lifespan is mostly lacking. The efficiency of genetic modification of cells also depends on the effective introduction of DNA vectors into the cells.

Adipose-derived mesenchymal stem cells (ADSCs) and skeletal muscle-derived satellite cells (MDSCs) are both derived from the mesoderm of adult pluripotent stem cells, though their pluripotency is less than that of ESCs, they have a high degree of proliferation, self-renewal and differentiation potential. Under different induction conditions, ADSCs and MDSCs can differentiate into bone, cartilage, muscle, tendon or fat mesodermal cells, and can also differentiate into nerve cells and hepatic oval cells [9], [10], [11], [12], [13]. Here, we characterize for the first time goat MDSCs (gMDSCs) isolated and cultured in vitro in terms of their proliferation capacity, morphologic appearance and uptake of exogenous DNA after transfection. The method for the in vitro separation and culture of Arbas cashmere goat ADSCs (gADSCs) has been previously published [14].

The present study aimed to establish a nuclear transfer technology system for Arbas cashmere goats and to investigate the use of gADSCs and gMDSCs as cell sources for SCNT compared with goat fetal fibroblast cells (gFFCs), including their proliferation capacity, pluripotency, transfection efficiency and capacity to support full term development of SCNT embryos after additive gene transfer or homologous recombination. The study’s aim of using ADSCs and MDSCs for SCNT mediated transgenesis in Arbas cashmere goats was achieved.

## Materials and Methods

### Ethics Statement

All studies adhered to procedures consistent with the International Guiding Principles for Biomedical Research Involving Animals issued by the Council for the International Organizations of Medical Sciences and were approved by the Institutional Animal Care and Use Committee of Inner Mongolia University. The owner of the YiWei White Cashmere Goat Farm also gave permission for the study.

### Materials

Arbas white cashmere goat fetuses (40 days) were obtained from the Experimental Animal Center of Inner Mongolia University. Females were maintained under pathogen-free conditions.

### Isolation and Identification of gMDSCs

gMDSCs were isolated using the method we have described previously [15]. Briefly, skeletal muscle from the leg was cut into 1–2 mm^3^ fragments, digested with 0.1% type I collagenase (C0130; Sigma–Aldrich, St. Louis, MO) in phosphate buffered saline (PBS) (14190-250; Invitrogen Corp., Carlsbad, CA ) for 1 h at 37°C in a water bath with shaking, and centrifuged at 150 *g* for 5 min. The supernatant was discarded and the pellet was resuspended in 0.25% trypsin (25200-056; Invitrogen Corp., Carlsbad, CA) and incubated for 20 min at 37°C. Fetal bovine serum (FBS) (12664-025; Invitrogen Corp., Carlsbad, CA) was added to the pellet, the mixture was centrifuged, and the pellet was resuspended in growth medium (Dulbecco’s modified Eagle’s medium [DMEM]/F12 [11320-082; Invitrogen Corp., Carlsbad, CA] containing 20% FBS, 10% horse serum [HS] [26050-088; Invitrogen Corp., Carlsbad, CA] and 1% penicillin/streptomycin [15140-122; Invitrogen Corp., Carlsbad, CA]). After repeated pipetting, the cells were passed through a 200 mesh sieve and centrifuged (150 *g* for 5 min).

The cells were plated in six-well plates coated with 0.1% gelatin (53028; Sigma–Aldrich, St. Louis, MO) at a density of 1 × 10^6^/well. gMDSCs were purified using the differential adhesion method and cultured in growth medium.

gMDSCs (1 × 10^4^ cells/well) were seeded in 24-well plates. The cells were fixed with 4% paraformaldehyde (16005; Sigma–Aldrich, St. Louis, MO) at 80% confluence for 30 min, permeabilized with PBS containing 0.1% (vol/vol.) Triton X-100 (T8787; Sigma–Aldrich, St. Louis, MO) and incubated with 3% bovine serum albumin (BSA) (A2058; Sigma–Aldrich, St. Louis, MO) in PBS for 2 h. The cells were then incubated with primary detection antibodies; desmin (ab32362; Abcam, Cambridge, UK), sarcomeric alpha-actinin (ab9465; Abcam, Cambridge, UK), MyoD1 (ab64159; Abcam, Cambridge, UK), Myf5 (ab125301; Abcam, Cambridge, UK) and PAX7 (ab34360; Abcam, Cambridge, UK) were diluted with 2% BSA to 1/200 at room temperature for 1 h. After washing in PBS, the cells were incubated with a mixture of fluorescein isothiocyanate (FITC)-conjugated goat anti-rabbit secondary antibodies (ab97050; Abcam, Cambridge, UK) and DAPI (D9542; Sigma–Aldrich, St. Louis, MO). The primary antibody was replaced with PBS for a negative control. Cell staining was viewed under a confocal microscope (A1; Nikon, Tokyo, Japan).

### gMDSCs Freeze–thaw and Growth Curve

gMDSCs at different passage numbers were mixed with a freezing protective agent (10% DMEM/F12+10% dimethyl sulfoxide [DMSO] [D2650; Sigma–Aldrich, St. Louis, MO] +80% HS) at 0.5 × 10^6^ cells/mL at –80°C for 24 h, and stocked in liquid nitrogen; before use, they were thawed quickly at 37°C. Cells at passage 50 were used to obtain growth curves. The cells were adjusted to 1 × 10^4^ cells/well and seeded in 24-well plates. Beginning the next day, cells were harvested from three wells for cell counting, continuing daily for 8 days to generate a growth curve.

### Apoptosis of gADSCs and gMDSCs in vitro

Fiftieth passage gADSCs and gMDSCs were washed twice with cold PBS and then cells at a concentration of 1 × 10^6^/mL were resuspended with 1× Binding Buffer, which was a constituent of the FITC Annexin V Apoptosis Detection Kit (556547; Becton Dickinson Biosciences, San Jose, CA), by centrifugation. One hundred microliters of cell suspension was taken into a centrifuge tube and 5 μL FITC-Annexin V and 5 μL propidium iodide (PI) were added with gentle vortexing, followed by incubation at room temperature for 15 min. Finally, another 400 ul 1× Binding Buffer was added to each tube. Apoptosis was detected by flow cytometry within 1 h.

### Neurogenic Differentiation and Identification

For neurogenic induction, 70% confluent gADSCs and gMDSCs were cultured in DMEM/F12 medium supplemented with 10 ng/mL epidermal growth factor (G5021; Promega, Madison, WI) plus 10 ng/mL basic fibroblast growth factor (bFGF) (G5071, Promega, Madison, WI) for 24 h. Subsequently, the medium was changed to DMEM/F12 supplemented with 1 mM β-mercaptoethanol (21985-023, Gibco, Gaithersburg, MD) and 10% FBS for 6 h for induction. The cells were then washed three times with PBS to remove β-mercaptoethanol and cultured in DMEM/F12 supplemented with 2% DMSO, 200 μM butylated hydroxyanisole (20021; Sigma–Aldrich, St. Louis, MO) and 20 ng/mL bFGF. When neuron-like cells were observed, the cells were stained with neuron-specific enolase (NSE) antibody (ab53025; Abcam, Cambridge, UK) and examined by immunofluorescence [11].

### Myogenic Induction and Identification

Fifth passage gADSCs and gMDSCs were seeded into six-well plates (1 × 10^5^ cells/mL) and cultured to 80% confluence; the medium was then replaced with myoblast induction medium. For gADSCs, the myoblast induction medium was DMEM/F12 supplemented with 10% FBS and 18 mg/mL hydrocortisone (H0135; Sigma–Aldrich, St. Louis, MO); for gMDSCs, the myoblast induction medium was DMEM/F12 supplemented with 10% HS. When thick, short or narrow, long club-shaped cells were observed, the cells were stained with 5 mg/L Hoechst 33342 (14533; Sigma–Aldrich, St. Louis, MO) to detect cell fusion. The cells were also fixed and stained using an antibody to detect myosin (ab108923; Abcam, Cambridge, UK), a marker of muscle differentiation, as previously described [16].

### Insulin-producing Cell Induction and Identification

gADSCs and gMDSCs were induced in vitro by incubation in DMEM Low Glucose (12320-032; Invitrogen Corp., Carlsbad, CA) containing 5% DMSO for 3 days, followed by culture in DMEM High-Glucose (10569-010; Invitrogen Corp., Carlsbad, CA) containing 10% FBS for 1 week. Cells cultured in DMEM/Low Glucose medium containing 10% FBS alone served as the control. After 10 days of culture, dithizone (D704; Sigma–Aldrich, St. Louis, MO) was used to confirm the induction of insulin-producing cells.

### G418 Sensitivity of gADSCs, gMDSCs and gFFCs

Second passage gADSCs, gMDSCs and gFFCs were incubated in 24-well plates (1 × 10^5^ cells/well). At 80% confluence, G418 (A1720; Sigma–Aldrich, St. Louis, MO) was added to triplicate wells at final concentrations of 100, 200, 300, 400, 500, 600, 700, 800, 900 or 1000 μg/mL. The medium was changed every 2 days for 14 days and the time at which all cells were dead was recorded. The optimal concentration for selection was defined as the lowest concentration at which all cells died within 5–6 days.

### Transfection and Selection

To investigate the effect of exogenous genes on gMDSC- and gMDSC-derived cloned embryo development, we used pDsRed2-1 (632405, Clontech, Santa Clara, CA) as an exogenous vector. gADSCs, gMDSCs and gFFCs were cultured in 24-well plates (1 × 10^5^ cells/well) for 24 h before transfection with pDsRed2-1 using Lipofectamine LTX and Plus Reagent (15338-100, Invitrogen Corp., Carlsbad, CA) according to the manufacturer’s protocol. The transient transfection efficiency was evaluated by flow cytometry. Similarly, gADSCs, gMDSCs and gFFCs were cultured in 100 mm plates for G418 and fluorescence screening, resulting in gADSCs–pDsRed2-1, gMDSCs–pDsRed2-1 and gFFCs–pDsRed2-1. After 10 days, colony forming efficiency was calculated. Neurogenic, myogenic and insulin-producing cell differentiation was induced in gADSCs–pDsRed2-1, gMDSCs–pDsRed2-1 and gFFCs–pDsRed2-1, respectively, as described above.

### Use of gADSCs–pDsRed2-1, gMDSCs–pDsRed2-1 and gFFCs–pDsRed2-1 as Nuclear Donors for SCNT

Ovaries were collected into 0.9% NaCl solution and dissected using a surgical scalpel to release cumulus–oocyte complexes (COCs). Well-formed COCs were identified by light microscopy on the basis of integrated cumulus cells, tight wrapping and having at least three layers of cumulus cells. Selected COCs were incubated in maturation medium (TCM199 [11150-059; Invitrogen Corp., Carlsbad, CA] +10% estrous goat serum [homemade] [17] +10 mM Hepes [H4034; Sigma–Aldrich, St. Louis, MO] +0.1 μg/mL 17β-estradiol [C13213100; Wako, Osaka, Japan] +10 μg/mL follicle stimulating hormone [F2293; Sigma–Aldrich, St. Louis, MO] +8 g/mL luteinizing hormone [L6420; Sigma–Aldrich, St. Louis, MO] +0.22 mg/mL sodium pyruvate [P4562; Sigma–Aldrich, St. Louis, MO]) and equilibrated at 38.5°C in an atmosphere of 5% CO_2_ in air for 18 h, after which the maturation rate (number of oocytes containing the first polar body/total oocytes) was calculated. The matured COCs were treated with 0.1% hyaluronidase (H3506; Sigma–Aldrich, St. Louis, MO) to remove cumulus cells. Cumulus-free oocytes were then enucleated by aspirating the first polar body and adjacent cytoplasm with a glass pipette in medium containing M199, 25 mM Hepes and 10% FBS. gADSCs–pDsRed2-1, gMDSCs–pDsRed2-1 and gFFCs–pDsRed2-1 were introduced into the enucleated oocytes under the zona pellucida and placed between 0.5 mm diameter platinum electrodes 1 mm apart in activation medium. Fusion/activation was induced with two direct current pulses (180 V/mm, 20 μs) using an Electro-Cell Manipulator 200 (BTX; San Diego, CA). The fusion rate was calculated.

### Reconstructed Embryo Activation and in vitro Culture

Reconstructed embryos was activated by 5 μM A23187 (C7522, Sigma–Aldrich, St. Louis, MO) for 5 min and then further activated with 2 mM 6-DMAP (D2629, Sigma–Aldrich) at 38.5°C under 5% CO_2_ in air for 3.5 h. The activated embryos were washed with development solution (TCM-199 supplemented with 5% FBS and 5 μg/mL gentamycin [G1264; Sigma–Aldrich, St. Louis, MO]). Cleavage and eight cell rates were determined at 48 h and the blastocyst rate was calculated at day 7–9. For detection of DsRed2 expression, embryos were examined using a fluorescence microscope. Red fluorescent protein expression was observed and compared between the three types of original cell.

### Real-time PCR

Expression of the DsRed2 gene in gADSCs–pDsRed2-1, gMDSCs–pDsRed2-1 and gFFCs–pDsRed2-1 embryos was investigated at the morula stage. Total mRNA was isolated from embryos and real-time PCR was performed on a MX4000 system (Stratagene, La Jolla, CA) using Brilliant SYBR Green QPCR Master Mix (Stratagene) and the primers listed in Table 1. The GAPDH gene was used as a housekeeping reference gene to normalize expression between the samples. Data from triplicate experiments were expressed relative to GAPDH, which was used to normalize for any differences in reverse transcriptase efficiency. Fold change in gene expression relative to the control was determined by the standard 2^–ΔΔCt^ method.

**Table 1:** Real-time PCR primers.

Gene	Primer sequences
GAPDH	5′-TTGTGATGGGCGTGAACC-3′
	5′-CCCTCCACGATGCCAAA-3′
DsRed2	5′-CCACTACCTGGTGGAGTTCAAG-3′
	5′-CTCGTTGTGGGAGGTGATGT-3′

### Western Blot Analysis

Aliquots of morula stage embryo lysates containing 45 μg of protein were separated by 12% sodium dodecyl sulfate polyacrylamide gel electrophoresis and transferred to nitrocellulose filters. The filters were blocked with TBST buffer (10 mM Tris-HCl [07066; Sigma–Aldrich, St. Louis, MO], pH 8.0, 0.15 M NaCl, 0.05% Tween 20 [P9416; Sigma–Aldrich, St. Louis, MO]) containing 5% skimmed milk (232100; BD Biosciences, San Jose, CA) and incubated with polyclonal antibodies specific for α-tubulin (ab125267; Abcam, Cambridge, UK) and DsRed2 (ab62341; Abcam, Cambridge, UK) overnight at 4°C. This was followed by the addition of horseradish peroxidase-conjugated anti-mouse IgG (ab6728; Abcam, Cambridge, UK) and enhanced chemiluminescence visualization of the bands. Bands were scanned using a GS-800 Calibrated Densitometer (Bio-Rad, Hercules, CA) and the intensities from each blot were quantified using ImageJ software (National Institutes of Health, Bethesda, MD).

### Epigenetic Modification of Embryos Derived from gADSCs–pDsRed2-1, gMDSCs–pDsRed2-1 and gFFCs–pDsRed2-1

gADSCs–pDsRed2-1, gMDSCs–pDsRed2-1 and gFFCs–pDsRed2-1 transgenic embryos at the two and four cell stage were studied using 5-methylcytosine (ab124936; Abcam Cambridge, UK), H4K5 (ab51997; Abcam, Cambridge, UK), H4K12 (ab104127; Abcam Cambridge, UK) and H3K18 (ab1191; Abcam Cambridge, UK) with an immunohistochemical method.

### Embryo Transplantation

#### Preparation of female Arbas cashmere goat recipients

The female Arbas cashmere goat recipients used in this study all met the following conditions. (1) They were adults and capable of recovering quickly after surgery. (2) They had already given birth, to ensure that the transgenic lambs were nurtured. (3) They were in natural estrus. (4) They were free from diseases of the reproductive system and underwent pest prevention treatment before transplantation.

#### Transgenic embryo transplantation and detection

Thirty-five recipients received transplanted transgenic embryos derived from gADSCs–pDsRed2-1, gMDSCs–pDsRed2-1 and gFFCs–pDsRed2-1. The goats were fasted for 20 h before surgery and were anesthetized before the procedure. Four to six embryos at the four to eight cell stage were transplanted into each recipients ipsilateral fallopian tubes. After surgery, the recipients were injected with an analeptic. The pregnancy rate was calculated after 150 days. Expression of DsRed2 in the skin of transgenic goats was detected by real-time PCR and western blot analysis as described above.

### Statistical Analysis

Every experiment was repeated at least three times. The data were presented as the mean and the standard error of the mean, and analyzed using SPSS Version 19.0 (IBM, Armonk, NY). Data were analyzed by multivariate two-way analysis of variance with post hoc tests to control for multiple comparisons. *P*<0.05 was considered statistically significant.

## Results

### Morphologic Characteristics, Identification and Growth of gMDSCs

**Figure 1:**
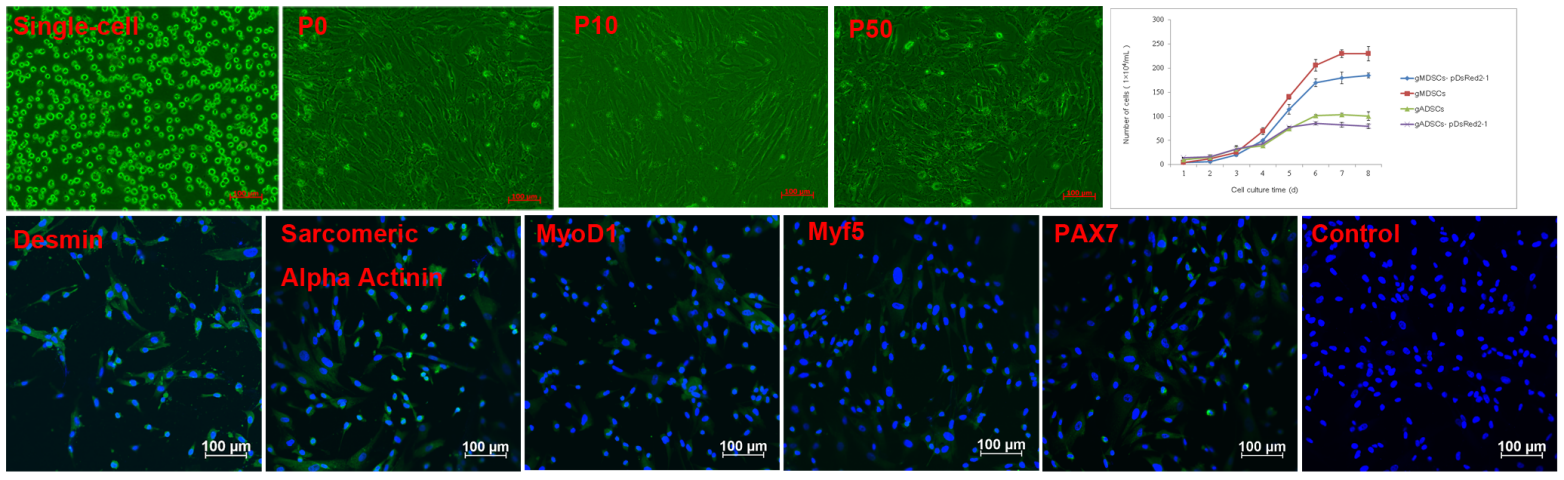
Morphologic changes, identification and growth of gMDSCs. Cell suspensions from skeletal muscle tissues were found to contain large numbers of small and rounded satellite cells. The vast majority of cells first transformed into spindle-shaped mononuclear cells after the second passage. Positive desmin, α-sarcomeric actinin, MyoD1, Myf5, and PAX7 immunostaining was visualized as green, whereas DAPI-positive nuclei were stained blue. gMDSCs and gMDSCs–pDsRed2-1 entered an exponential growth phase between days 2 and 3, and reached a growth plateau between days 6 and 7. gADSCs and gADSCs–pDsRed2-1 gave results similar to those for gMDSCs and gMDSCs–pDsRed2-1. gMDSCs, goat muscle-derived satellite cells; gADSCs, goat adipose-derived mesenchymal stem cells.

Cell suspensions from skeletal muscle tissues were found to contain a large number of small, rounded satellite cells (96%) and few large, mature, rod-shaped myotube cells (4%) on flow cytometry. The vast majority of cells first transformed into polygonally shaped cells and then into spindle-shaped mononuclear cells within 48 h, with abundant cytoplasm and a high cellular refractive index (Figure 1).

Positive Desmin, Sarcomeric Alpha Actinin, MyoD1, Myf5 and PAX7 immunostaining was visualized as green, whereas DAPI-positive nuclei were stained blue (Figure 1). Morphologic characteristic analysis combined with surface marker molecules confirmed that the cultured cells were gMDSCs and were suitable for further research.

gMDSCs entered an exponential growth phase between days 2 and 3, and reached a growth plateau between days 6 and 7. gMDSCs–pDsRed2-1 entered an exponential growth phase at 2 d and reached a growth plateau at 6 d. Compared with gMDSCs–pDsRed2-1, the growth rate of MDSCs was significantly higher, starting at 4 d (*P*<0.05) (Figure 1). The findings for gADSCs and gADSCs–pDsRed2-1 were similar to those for gMDSCs and gMDSCs–pDsRed2-1.

### Apoptosis of gADSCs, gADSCs–pDsRed2-1, gMDSCs and gMDSCs–pDsRed2-1

**Figure 2:**
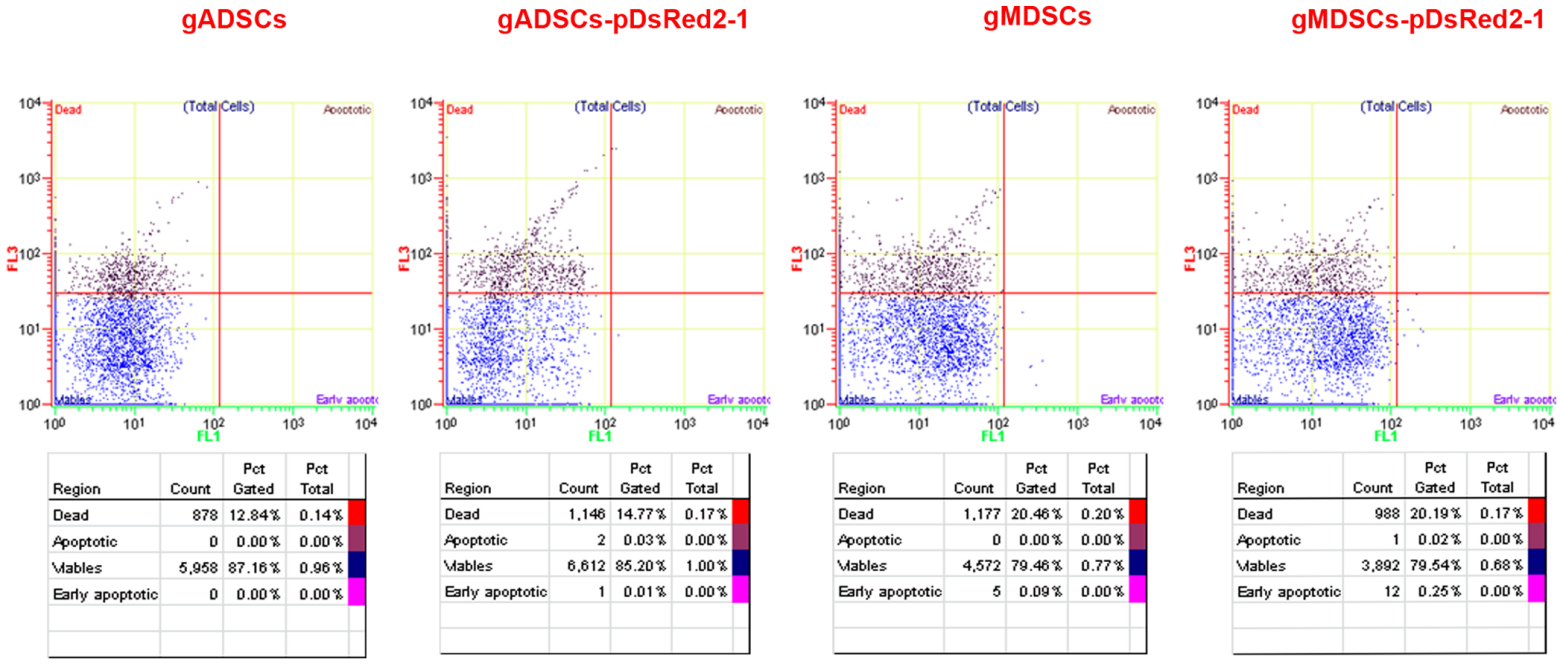
Apoptosis of gADSCs, gADSCs–pDsRed2-1, gMDSCs and gMDSCs–pDsRed2-1. The percentages of apoptotic and early apoptotic cells were 0.00% and 0.00%, and 0.00% and 0.09%, respectively, in 50th generation gADSCs and gMDSCs. The percentages of apoptotic and early apoptotic cells were 0.03% and 0.01%, and 0.02% and 0.25%, respectively, in 10th generation gADSCs–pDsRed2-1 and gMDSCs–pDsRed2-1. gADSCs, goat adipose-derived mesenchymal stem cells; gMDSCs, goat muscle-derived satellite cells.

The percentages of apoptotic and early apoptotic cells were 0.00% and 0.00%, and 0.00% and 0.09%, respectively, in 50th generation gADSCs and gMDSCs as demonstrated by FITC-Annexin V/PI assay. The percentages of apoptotic and early apoptotic cells were 0.03% and 0.01%, and 0.02% and 0.25%, respectively, in 10th generation gADSCs–pDsRed2-1 and gMDSCs–pDsRed2-1. The percentage of dead cells was 15–20% for each of the four types of cell (Figure 2).

### Induced Neuronal Differentiation and NSE Expression of gADSCs, gADSCs–pDsRed2-1, gMDSCs and gMDSCs–pDsRed2-1

**Figure 3:**
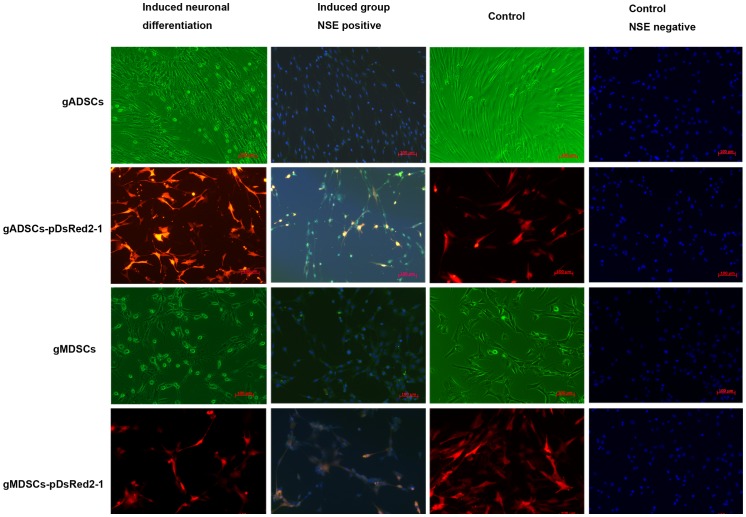
Neuronal differentiation of gADSCs, gADSCs–pDsRed2-1, gMDSCs and gMDSCs–pDsRed2-1. gADSCs, gADSCs–pDsRed2-1, gMDSCs and gMDSCs–pDsRed2-1 were altered morphologically by incubation in induction medium containing β-mercaptoethanol. The cytoplasm began to shrink, and after 2 h the cell bodies became conical, triangular or round in shape with multiple protrusions resembling axons, the ends of which were primary and secondary bifurcated dendrites. Immunohistochemistry showed that the cells were NSE *(green)* positive after 3 h. Uninduced control cells were morphologically unaltered and did not express NSE. gADSCs, goat adipose-derived mesenchymal stem cells; gMDSCs, goat muscle-derived satellite cells; NSE, neuron-specific enolase.

gADSCs and gMDSCs were altered morphologically by incubation in induction medium containing β-mercaptoethanol. After 30 min the cytoplasm began to shrink; after 2 h, the cell bodies became conical, triangular or round in shape with multiple protrusions resembling axons, the ends of which were primary and secondary bifurcated dendrites. Immunohistochemistry showed that the cells were NSE positive after 3 h. Uninduced gADSCs and control gMDSCs were morphologically unaltered and did not express NSE after 4 h. gADSCs–pDsRed2-1 and gMDSCs–pDsRed2-1 exhibited a similar induction pattern, suggesting that the integrated exogenous genes did not influence the induction of neuronal differentiation of gADSCs–pDsRed2-1 or gMDSCs–pDsRed2-1 (Figure 3).

### Myogenic Induction of gADSCs, gADSCs–pDsRed2-1, gMDSCs and gMDSCs–pDsRed2-1

**Figure 4:**
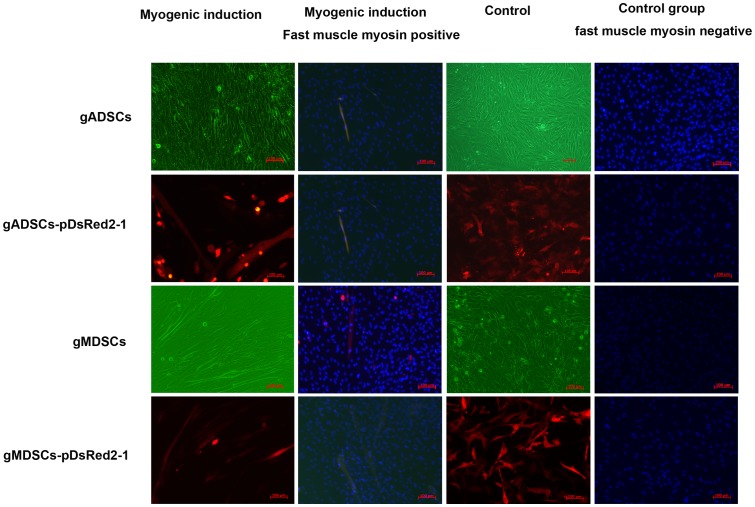
Myogenic induction of gADSCs, gADSCs–pDsRed2-1, gMDSCs and gMDSCs–pDsRed2-1. gMDSCs and gMDSCs–pDsRed2-1 began to fuse after 2 d and myotube cells increased markedly. gADSCs and gADSCs–pDsRed2-1 began to integrate 5 d after myogenic induction, leading to short myotube cell formation. DAPI staining revealed the presence of multiple nuclei in the same myotube, and immunohistochemical staining demonstrated that the cells were fast muscle myosin *(green)* positive; control (uninduced) cell were fast muscle myosin negative. gADSCs, goat adipose-derived mesenchymal stem cells; gMDSCs, goat muscle-derived satellite cells.

Forty-eight hours after the addition of myogenic induction medium, gMDSCs and gMDSCs–pDsRed2-1 had begun to fuse, and gradually became arranged in parallel with each other oriented in a single direction to form short, thick, multicore myotube cells. As time passed, the cell density increased, the integration between the cells became more extensive, and the number and length of the myotube cells increased markedly. gADSCs and gADSCs–pDsRed2-1 began to integrate 5 d after myogenic induction, leading to obvious myotube cell formation (Figure 4). DAPI staining revealed the presence of multiple nuclei in the same myotube, and immunohistochemical staining demonstrated that the cells were fast muscle myosin positive; control (uninduced) cells were fast muscle myosin negative (Figure 4). These results indicate that gADSCs, gADSCs–pDsRed2-1, gMDSCs and gMDSCs–pDsRed2-1 can be induced to differentiate into myogenic cells.

### Differentiation and Confirmation of Insulin-producing Cells

**Figure 5:**
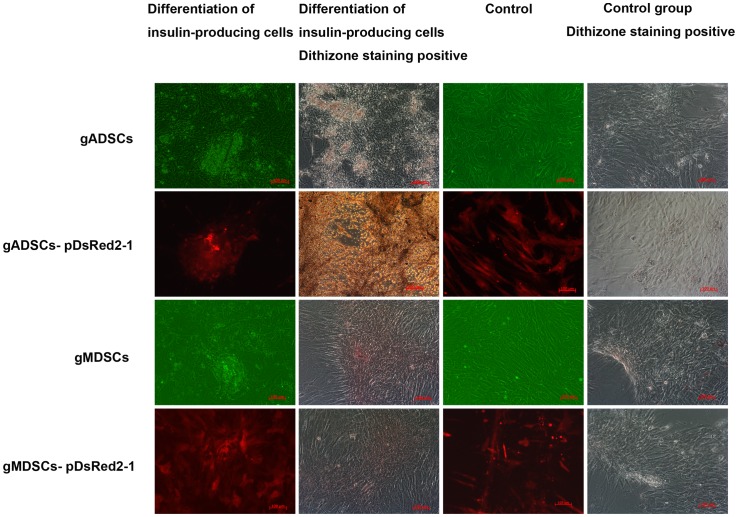
Differentiation and confirmation of insulin-producing cells. gADSCs, gADSCs–pDsRed2-1, gMDSCs and gMDSCs–pDsRed2-1 exhibited intensive cell growth. There were irregular cell clusters in the control group, and the cells were divergent and fusiform. Cell masses were scarlet in the induced group after dithizone staining but remained unstained in the control group. Insulin was detected in fusiform cells in the induced group, but the control group lacked insulin. gADSCs, goat adipose-derived mesenchymal stem cells; gMDSCs, goat muscle-derived satellite cells.

Three days after induction of gADSCs, gADSCs–pDsRed2-1, gMDSCs and gMDSCs–pDsRed2-1 in DMEM/Low Glucose, the nucleus: cytoplasm ratio was altered and the nuclei became significantly larger (Figure 5). At day 5, the induced cells exhibited intensive cell growth. In the control group, there were irregular cell clusters and the cells were divergent and fusiform. Cell masses were scarlet in the induced group after dithizone staining, but remained unstained in the control group. Insulin was detected in fusiform cells in the induced group, but the control group lacked insulin (Figure 5).

### G418 Sensitivity of gADSCs, gMDSCs and gFFCs

**Figure 6:**
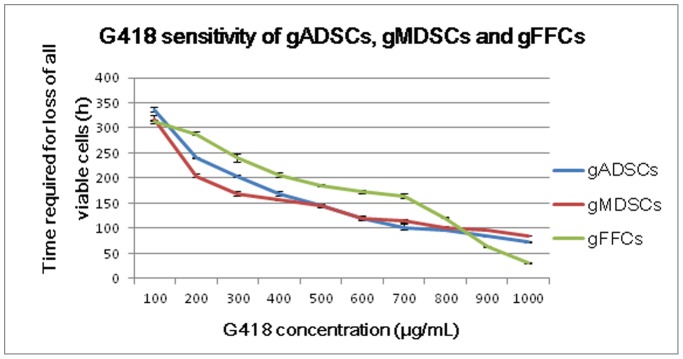
G418 sensitivity of gADSCs, gMDSCs and gFFCs. For gADSCs, 400 μg/mL G418 was detected by day 7 and was selected as the optimal screening concentration for transgenic cell clones. For gMDSCs, 300 μg/mL G418 was selected as the optimal screening concentration. For gFFCs 700 μg/mL G418 was selected. gADSCs, goat adipose-derived mesenchymal stem cells; gMDSCs, goat muscle-derived satellite cells; gFFCs, goat fetal fibroblast cells.

The sensitivity of gADSCs, gMDSCs and gFFCs to G418 increased gradually with concentration in the range 100 μg/mL to 1000 μg/mL. For gADSCs, 400 μg/mL G418, the concentration at which no viable cells were detected by day 7, was selected as the optimal screening concentration for transgenic cell clones; 200 μg/mL, the concentration at which no viable cells were detected by day 10, was selected as the maintenance concentration. For gMDSCs, 300 μg/mL was selected as the optimal screening concentration; 150 μg/mL was the maintenance concentration. Based on identical criteria, the optimal screening and maintenance G418 concentrations for gFFCs were identified as 700 μg/mL and 350 μg/mL, respectively (Figure 6).

### In vitro G418 Screening of gADSCs–pDsRed2-1, gMDSCs–pDsRed2-1 and gFFCs–pDsRed2-1 in Monoclonal Culture

No significant differences were detected among the transient transfection efficiencies of gADSCs, gMDSCs and gFFCs (12%, 13.7% and 12.8%, respectively) (*P*>0.05) 48 h after transfection. The rates of neomycin resistant monoclonal colony formation and red fluorescence in gADSCs–pDsRed2-1, gMDSCs–pDsRed2-1 and gFFCs–pDsRed2-1 were 78.26%, 85.67% and 50.00%, respectively, after G418 screening (*P*<0.01) (Table 2).

**Table 2:** Comparison of fluorescent clone formation after pDsRed2-1 plasmid transfection of different cell lines.

Cell lines	1	2	3	Total	Standard deviations
gADSCs^a^-pDsRed2-1	18/23	20/25	16/21	54/69	2%
gMDSCs^b^-pDsRed2-1	162/181	192/231	190/223	544/635	3%
gFFCs^c^-pDsRed2-1	15/28	17/38	17/32	49/98	5%

Data are the means of the number of fluorescent cell populations observed in three Petri dishes. Formation rate = number of fluorescent cell populations in one Petri dish/total number of clones.

a Goat adipose-derived mesenchymal stem cells.

b Goat muscle-derived satellite cells.

c Goat fetal fibroblast cells.

### Nuclear Transfer and Transgenic Embryonic Development of gADSCs–pDsRed2-1, gMDSCs–pDsRed2-1 and gFFCs–pDsRed2-1

One thousand, seven hundred and fifty-five COCs were cultured in vitro following nuclear transfer; among these, 1322 oocytes matured in vitro (75.33% contained the first polar body). For gADSCs–pDsRed2-1, of 551 oocyte–donor cell complexes (91.7%) surviving after micromanipulation, 467 (84.75%) were electrofused successfully. Of these, 410 transgenic embryos (87.79%) had cleaved after 48 h, 132 transgenic embryos (28.27%) reached the eight cell phase and 49 (10.49%) developed into transgenic blastocysts after 7–9 d (Table 3). For gMDSCs–pDsRed2-1, of 466 oocyte–donor cell complexes (91.7%) surviving after micromanipulation, 427 (92.83%) were electrofused successfully. Of these, 365 transgenic embryos (85.48%) had cleaved after 48 h, 114 transgenic embryos (26.70%) reached the eight cell phase and 18 (11.18%) developed into transgenic blastocysts after 7–9 d (Table 3). For gFFCs–pDsRed2-1, 208 oocyte–donor cell complexes survived after micromanipulation and 151 (72.60%) were successfully electrofused. Of these, 116 transgenic embryos (76.82%) had cleaved after 48 h, 38 transgenic embryos (25.17%) reached the eight cell phase and 15 (9.93%) developed into blastocysts after 7–9 d (Table 3).

**Table 3:** Maturation and development of cloned embryos derived from gADSCs^a^–pDsRed2-1, gMDSCs^b^–pDsRed2-1 and gFFCs^c^–pDsRed2-1 in vitro.

	Group	Number of cumulus–oocyte complexes	Number of mature embryos	Number of surviving embryos	Number of embryos exhibiting convergence	Number of embryos exhibiting cleavage	Number of eight-cell embryos	Number of blastocysts
gADSCs–pDsRed2-1			(Maturity rate)	(Survival rate)	(Convergence rate)	(Cleavage rate)	(Eight-cell rate)	(Blastocyst rate)
	A	237	185	167	145	131	43	15
			(78.06%)	(90.27%)	(86.82%)	(90.34%)	(29.66%)	(10.34%)
	B	281	205	188	152	130	41	15
			(72.95%)	(91.70%)	(80.85%)	(85.52%)	(26.97%)	(9.86%)
	C	263	210	196	170	149	48	19
			(79.85%)	(93.33%)	(86.73%)	(87.65%)	(28.24%)	(11.18%)
	Total	781	600	551	467	410	132	49
			(76.82%)	(91.83)	(84.75)	(87.79%)	(28.27%)	(10.49%)
	Sd^d^		3.58	1.53	3.42	2.42	1.35	0.67
gMDSCs–pDsRed2-1	A	231	172	162	155	133	38	18
			(74.46%)	(94.18%)	(90.12%)	(85.81%)	(24.51%)	(11.61%)
	B	281	203	188	171	149	49	16
			(72.24%)	(92.61%)	(90.96%)	(87.13%)	(28.66%)	(9.37%)
	C	161	122	110	101	83	27	9
			(75.78%)	(90.16%)	(91.82%)	(82.18%)	(26.73%)	(8.91%)
	Total	673	497	460	427	365	114	43
			(73.85%)	(92.56%)	(92.83%)	(85.48%)	(26.70%)	(10.07%)
	Sd^d^		1.79	2.03	0.85	2.56	2.08	1.44
gFFCs–pDsRed2-1	A	94	68	61	45	41	13	6
			(72.34%)	(89.71%)	(73.77%)	(91.11%)	(28.88%)	(13.33%)
	B	87	71	68	60	54	19	7
			(81.61%)	(95.77%)	(88.24%)	(90.00%)	(31.67%)	(11.67%)
	C	120	88	79	56	49	17	5
			(73.33%)	(89.77%)	(70.89%)	(87.50%)	(30.36%)	(8.93%)
	Total	301	227	208	161	144	49	18
			(75.42%)	(91.63%)	(77.40%)	(89.44%)	(30.43%)	(11.18%)
	Sd^d^		5.09	3.48	9.30	1.85	1.40	2.22

a Goat adipose-derived mesenchymal stem cells.

b Goat muscle-derived satellite cells.

c Goat fetal fibroblast cells.

d Standard deviation.

The transgenic cloned embryos derived from gADSCs–pDsRed2-1 or gMDSCs–pDsRed2-1 exhibited approximately 20% and 10% higher convergence and cleavage rates than gFFCs–pDsRed2-1 respectively. No significant differences in the eight cell and blastocyst rates were detected.

**Figure 7:**
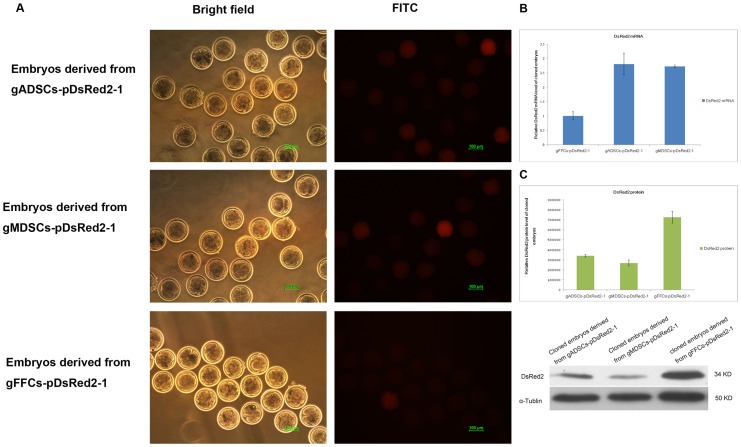
DsRed2 expression in cloned transgenic embryos derived from gADSCs–pDsRed2-1, gMDSCs–pDsRed2-1 and gFFCs–pDsRed2-1. A Transgenic embryos derived from gADSCs–pDsRed2-1, gMDSCs–pDsRed2-1 and gFFCs–pDsRed2-1. B DsRed2 mRNA expression of cloned embryos derived from gADSCs or gMDSCs was about 2.71 times greater than that of embryos derived from gFFCs–pDsRed2-1 (*P*<0.01), but the DsRed2-1 mRNA expression of cloned embryos derived from gADSCs was similar to that of embryos derived from gMDSCs (*P*>0.05). C DsRed2 protein expression of cloned embryos derived from gFFCs–pDsRed2-1 was 2.13 times greater than that of embryos derived from gADSCs–pDsRed2-1 and 2.71 greater than that of embryos derived from gMDSCs–pDsRed2-1 (*P*<0.01). gADSCs, goat adipose-derived mesenchymal stem cells; gMDSCs, goat muscle-derived satellite cells; gFFCs, goat fetal fibroblast cells.

gADSCs–pDsRed2-1, gMDSCs–pDsRed2-1 and gFFCs–pDsRed2-1 embryos in different phases were selected randomly for fluorescence examination. All transgenic embryos expressed red fluorescent protein but the levels varied between embryos at the same phase, with higher levels detected in embryos derived from gMDSCs–pDsRed2-1 (Figure 7A) compared with those derived from gADSCs–pDsRed2-1 or gFFCs–pDsRed2-1 (48.88% vs 35.26% and 31.15%, respectively) (*P*<0.01) (Figure 7A).

Real-time quantitative PCR analysis showed that the DsRed2 mRNA expression of cloned embryos derived from gADSCs or gMDSCs was about 2.71 times greater than that of embryos derived from gFFCs–pDsRed2-1 (*P*<0.01), but DsRed2-1 mRNA expression of cloned embryos derived from gADSCs was similar to that of embryos derived from gMDSCs (*P*>0.05) (Figure 7B).

By contrast, western blot analysis showed that the DsRed2 protein expression of cloned embryos derived from gFFCs–pDsRed2-1 was 2.13 times greater than that of embryos derived from gADSCs–pDsRed2-1 and 2.71 greater than that of embryos derived from gMDSCs–pDsRed2-1 (*P*<0.01) (Figure 7C). DsRed2 protein was detected as a single band in embryos derived from both types of cell.

### Expression of 5-methylcytosine, H4K5, H4K12 and H3K18 in Transgenic Embryos

**Figure 8:**
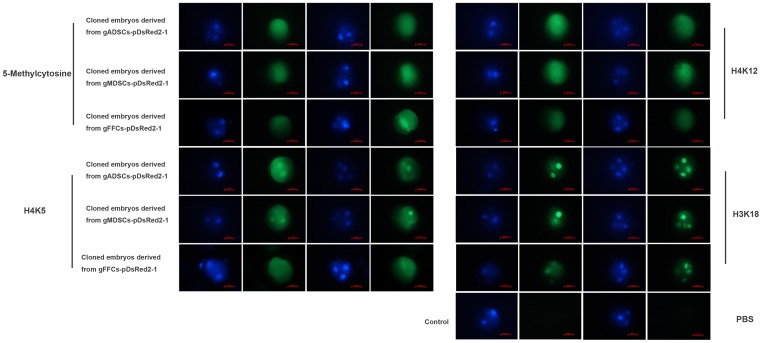
5-Methylcytosine, H4K5, H4K12 and H3K18 expression in embryos obtained by somatic cell nuclear transfer. Methylation levels assessed by 5-methylcytosine staining remained unchanged in cloned embryos derived from gADSCs–pDsRed2-1, gMDSCs–pDsRed2-1 and gFFCs–pDsRed2-1. H4K5 expression in the nucleus was higher in cloned embryos derived from gADSCs–pDsRed2-1 or gMDSCs–pDsRed2-1 than in those derived from gFFCs–pDsRed2-1. H4K12 expression in the nucleus and cytoplasm did not differ among the three types of embryo. H3K18 was highly expressed in the nucleus during both the two and the four cell stage. gADSCs, goat adipose-derived mesenchymal stem cells; gMDSCs, goat muscle-derived satellite cells; gFFCs, goat fetal fibroblast cells.

At different time points, the pattern of 5-methylcytosine, H4K5, H4K12 and H3K18 expression in SCNT embryos was examined by immunostaining (Figure 8). Levels of methylation assessed by 5-methylcytosine staining remained unchanged in cloned embryos derived from gADSCs–pDsRed2-1, gMDSCs–pDsRed2-1 or gFFCs–pDsRed2-1. H4K5 was more highly expressed in the nucleus in embryos derived from gADSCs–pDsRed2-1 or gMDSCs–pDsRed2-1 than in those derived from gFFCs–pDsRed2-1. H4K12 expression in the nucleus and cytoplasm in did not differ between the three types of embryo. H3K18 was highly expressed in the nucleus during both the two and the four cell stage.

### Transgenic Embryo Transplantation and Detection of Transgenic Goats

**Figure 9:**
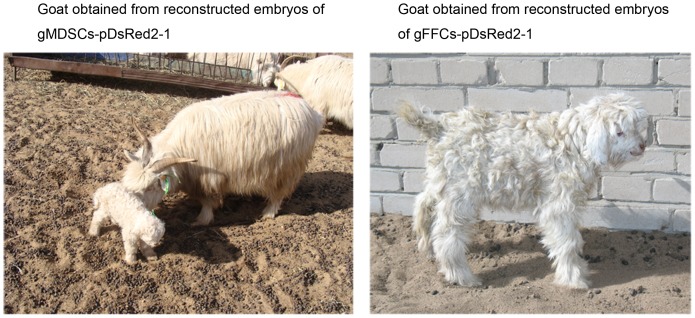
Transgenic goats obtained from reconstructed embryos derived from gMDSCs–pDsRed2-1 (left) and gFFCs–pDsRed2-1 (right). gMDSCs, goat muscle-derived satellite cells; gFFCs, goat fetal fibroblast cells.

In the gADSCs–pDsRed2-1, gMDSCs–pDsRed2-1 and gFFCs–pDsRed2-1 groups, two, three and one recipient, respectively, became pregnant. Zero, two and one cloned goat, respectively, were obtained (Figure 9).

## Discussion

At present, fibroblasts are predominantly used as the nuclear donor cells in cashmere goat transgenic cloning [6], [18], [19], but these cells have the significant disadvantages of limited number of passages and low survival rate after transfection, and have become a bottleneck in the breeding of new transgenic varieties.

Comprehensive analysis of well-defined cell lines is beneficial for the selection of appropriate cell types for use in advanced transgenic strategies to improve the production efficiency of large animal models using primary cells [18], [20], [21], [22]. The gADSCs and gMDSCs generated in this study proliferated rapidly in vitro and exhibited common properties of stem cells (i.e. self-renewal and multipotential differentiation) [3], [23], [24], [25], [26]. Furthermore, no marked signs of aging or abnormal apoptosis were detected in gADSCs or gMDSCs after the 65th passage in vitro, whereas abnormal karyotypes and marked signs of aging were apparent in gFFCs after the 15th passage in vitro. Cell growth curves showed that gADSCs, gADSCs–pDsRed2-1, gMDSCs and gMDSCs–pDsRed2-1 all experienced a latent period, a logarithmic growth phase and a plateau during growth in in vitro culture, indicating that the integrated exogenous genes did not influence proliferation efficiency. After numerous passages, reduced cell morphologic diversity might contribute to fibroblast-like cell proliferation and different cell cycles under culture conditions.

The efficiency of SCNT for the production of cloned animals in large batches is critically dependent on the source of the donor nucleus. In the present study, exogenous genes were transfected into gADSCs and gMDSCs using the liposome method. The transient transfection efficiencies of these two cell types were similar at 48 h after transfection, whereas the clone formation efficiency of gADSCs–pDsRed2-1 and gMDSCs–pDsRed2-1 was approximately 30% higher (*P*<0.01) than that of gFFCs–pDsRed2-1 on G418 screening after 10 d; thus, the accuracy of transgenic cell screening was greatly improved [27]. Furthermore, we observed low cytotoxicity and only marginal alterations of gADSC and gMDSC morphology after transfection, with no effects on cell properties such as response to chemicals and pattern of gene expression. The sensitivity of mammalian cells to G418 concentration is related to the species and to cell type and growth state. Compared with gFFCs, gMDSCs are more sensitive to G418, thereby reducing the quantity of G418 required for selection. Furthermore, the exogenous genes did not affect the growth rate or pluripotency of the transfected cells.

Low cloning efficiency has hampered the production of cloned animals. Several reports have indicated that the type of donor cell can affect the birth rate. In mice, an appropriate interaction between cell type and genotype can improve cloning efficiency [28]. Coordination of donor cell type and cell cycle stage can maximize the overall cloning efficiency [29]. In buffalos, cumulus cells are a more efficient nuclear donor for SCNT than fibroblasts [30]. In rabbits, embryos reconstructed with fresh cumulus cells have a more efficient developmental potential than those reconstructed with fetal fibroblasts both in vivo and in vitro [31]. However, comparisons show that adult cells of any type are inferior to fetal fibroblasts in terms of reconstructed embryo development.

Our results reconfirmed the fact that the type of donor somatic cell is critical for determining developmental competence. Moreover, these results further confirm that gADSCs and gMDSCs are more efficient as donor cells in SCNT for the cloning of Arbas cashmere goats. The convergence and cleavage rates of transgenic embryos derived from gADSCs or gMDSCs were higher than those of transgenic embryos derived from gFFCs after in vitro culture, whereas the eight cell and blastocyst rates were similar. Studies conducted in transgenic pigs by Faast et al. [32] in 2006 demonstrated that the use of pig bone marrow stem cells as SCNT donor cells did not improve the cleavage rate of cloned embryos compared with fibroblasts derived from the same pig, whereas the blastocyst rate was twofold greater than that of cloned embryos derived from fibroblasts. These data are not in accordance with those of our study and it can be speculated that this difference may be attributed to species differences and different epigenetic patterns affecting production.

Elevating histone acetylation levels improves the rate of development to blastocyst and to term and even the efficiency of ESC derivation from SCNT blastocysts [33], [34]. Because Lys-5 is the last lysine to be acetylated, acetylated H4K5 reflects hyperacetylated histone H4, and this is correlated with a transcriptionally permissive state of chromatin [35] that may be a necessary step for embryonic genome activation occurring at the two cell stage. High levels of H4K5 histone acetylation in embryos derived from gADSCs or gMDSCs may be another factor in their high cloning efficiency. Rogers et al. [36] verified that homologous recombination depends on donor cells. This underlines the importance of the characterization of donor cell cultures provided for genetic modification and SCNT.

This study is the first to report successful cloning using gADSCs and gMDSCs from Arbas cashmere goats. These goats can be used as a large animal model in transgenic research.

## Conclusions

gADSCs and gMDSCs were found to exhibit better proliferation rates, growth capacity, transfection efficiency and convergence and cleavage after SCNT compared with gFFCs. gADSCs and gMDSCs are highly suitable for additive gene transfer and the subsequent production of genetically modified goats by SCNT.
